# Discovery of Terpenes as Novel HCV NS5B Polymerase Inhibitors via Molecular Docking

**DOI:** 10.3390/pathogens12060842

**Published:** 2023-06-18

**Authors:** Tomasz M. Karpiński, Marcin Ożarowski, Pedro J. Silva, Mark Stasiewicz, Rahat Alam, Abdus Samad

**Affiliations:** 1 Chair and Department of Medical Microbiology, Poznań University of Medical Sciences, Rokietnicka 10, 60-806 Poznań, Poland; 2 Department of Biotechnology, Institute of Natural Fibres and Medicinal Plants—National Research Institute, Wojska Polskiego 71b, 60-630 Poznań, Poland; 3 FP-I3ID/Fac. de Ciências da Saúde, Universidade Fernando Pessoa, 4200-150 Porto, Portugal; 4 UCIBIO@REQUIMTE, BioSIM, Departament of Biomedicine, Faculty of Medicine, Universidade do Porto, 4200-319 Porto, Portugal; 5 Research Group of Medical Microbiology, Chair and Department of Medical Microbiology, Poznań University of Medical Sciences, Rokietnicka 10, 60-806 Poznań, Poland; 6 Biological Solution Centre (BioSol Centre), Farmgate, Dhaka 1215, Bangladesh

**Keywords:** hepatitis, terpenoids, treatment, in silico, molecular dynamics, new drugs, plant compounds

## Abstract

Hepatitis C virus (HCV) is a dangerous virus that is responsible for a large number of infections and deaths worldwide. In the treatment of HCV, it is important that the drugs are effective and do not have additional hepatotoxic effects. The aim of this study was to test the in silico activity of 1893 terpenes against the HCV NS5B polymerase (PDB-ID: 3FQK). Two drugs, sofosbuvir and dasabuvir, were used as controls. The GOLD software (CCDC) and InstaDock were used for docking. By using the results obtained from PLP.Fitness (GOLD), pKi, and binding free energy (InstaDock), nine terpenes were finally selected based on their scores. The drug-likeness properties were calculated using Lipinski’s rule of five. The ADMET values were studied using SwissADME and pkCSM servers. Ultimately, it was shown that nine terpenes have better docking results than sofosbuvir and dasabuvir. These were gniditrin, mulberrofuran G, cochlearine A, ingenol dibenzoate, mulberrofuran G, isogemichalcone C, pawhuskin B, 3-cinnamyl-4-oxoretinoic acid, DTXSID501019279, and mezerein. Each docked complex was submitted to 150 ns-long molecular dynamics simulations to ascertain the binding stability. The results show that mulberrofuran G, cochlearine A, and both stereoisomers of pawhuskin B form very stable interactions with the active site region where the reaction product should form and are, therefore, good candidates for use as effective competitive inhibitors. The other compounds identified in the docking screen either afford extremely weak (or even hardly any) binding (such as ingenol dibenzoate, gniditrin, and mezerein) or must first undergo preliminary movements in the active site before attaining their stable binding conformations, in a process which may take from 60 to 80 ns (for DTXSID501019279, 3-cinnamyl-4-oxoretinoic acid or isogemichalcone C).

## 1. Introduction

The hepatitis C virus (HCV) is a blood-borne pathogen that can lead to the development of chronic liver disease. HCV belongs to the genus Hepacivirus and the family Flaviviridae. It has positive-sense single-stranded RNA, which encodes structural (core, E1 and E2) and nonstructural proteins (p7, NS2, NS3, NS4A, NS4B, NS5A and NS5B) [1]. The worldwide prevalence of HCV is about 1.8%, and the highest prevalence (7.1%) of HCV is found in regions of Africa [2]. The World Health Organization (WHO) estimates that 58 million people have chronic HCV infection. Almost 300,000 deaths annually may be attributed to HCV [3]. HCV can lead to chronic hepatitis, cirrhosis, and hepatocellular carcinoma (HCC). Extrahepatic manifestations include polyarthritis, systemic vasculitis, lymphoproliferative diseases, renal disorders, type 2 diabetes, and pancreatic cancer [4,5]. There is no HCV vaccine currently available on the market, and access to diagnostics and treatment in many countries is low [3].

One of the molecular targets for drugs is the NS5B protein. NS5B is an RNA-dependent RNA polymerase. It is responsible for the formation of the viral RNA replication complex and the synthesis of viral RNA [6]. Currently, only two NS5B inhibitors are approved for use, sofosbuvir and dasabuvir [1,7], with many substances currently being evaluated in clinical trials [1]. However, serious problems include the development of drug resistance, insufficient anti-HCV potency, or adverse reactions [6].

Terpenes are natural compounds found mainly in plants that we encounter every day. Terpenes are classified mainly as monoterpenes (C10), sesquiterpenes (C15), diterpenes (C20), and triterpenes (C30). The most famous are the anti-microbials limonene, β-caryophyllene, and α-pinene, the antivirals cineol and borneol, the anti-inflammatory (-)—linalool and linalyl acetate, and the anti-malarial artemisinin. In addition, many herbs and medicinal oils include carvacrol, limonene, linalool, 1,8-cineole, eugenol, and/or menthol [8,9]. A number of articles highlight the antiviral effects of terpenes [10,11,12,13]. We were most inspired by the publication by Kong et al. [14], who presented that two terpenes, oleanolic acid and ursolic acid, inhibited the activity of the NS5B polymerase. Therefore, the aim of our in silico research was to find potential new NS5B HCV drugs among terpenes.

## 2. Materials and Methods

### 2.1. Ligands and Receptor

A collection of 1893 terpenes was obtained from the PubChem online database (https://pubchem.ncbi.nlm.nih.gov/), accessed on 20 September 2022. The 3D structures were downloaded in the SDF format. Additionally, the structures of 2 drugs inhibiting NS5B HCV, sofosbuvir and dasabuvir, were utilized.

The structure of the hepatitis C virus polymerase NS5B with an HCV inhibitor (PDB-ID: 3FQK at a resolution of 2.2 Å) was obtained from the RCSB Protein Data Bank (https://www.rcsb.org/), accessed on 20 September 2022.

### 2.2. Docking

For docking, GOLD software 2022 (The Cambridge Crystallographic Data Centre, UK) [15] and InstaDock (India) [16] were used. Firstly, GOLD was applied for the virtual screening of terpene ligands and the NS5B protein. Hydrogens were added to the PDB:3FQK structure [17], with water and crystallized ligands being removed. The position of the crystalized ligand (HCV-796) was selected as the docking site. “Early termination” was disabled in the software, and docking poses were sorted according to their ChemPLP scores. The higher the ChemPLP score, the better the result [15]. Based on this score, 20 terpenes were selected for further analysis. Next, the 20 selected terpenes, sofosbuvir, and dasabuvir were tested using GOLD as flexible docking. The PLP.fitness scores were obtained from this portion of the study [18]. The 20 selected terpenes, sofosbuvir, and dasabuvir were also tested using InstaDock to obtain the binding free energy, pKi, and ligand efficiency. InstaDock is a simple tool that automatizes the virtual screening docking process [16]. Finally, all the terpenes were also docked using AutoDock Vina [19,20]. The docking area was selected using the structures of PDB:3FQK (bound to the HCV-796 inhibitor) and PDB:4WTF [21] (an engineered version of the polymerase, which contains bound sofosbuvir). After the superposition of the structures, a box with sides 8 Å away from the atoms of any of the two ligands was built, and this box (with dimensions 34 × 34 × 29 Å) was used as the docking area of the ligand to the PODB:3FQK structure. Given the results of the PLP.Fitness (GOLD), InstaDock binding free energy (InstaDock), and the VINA score, eight terpenes were selected for docking pose stability analysis through molecular dynamics simulations. 

### 2.3. In Silico Drug-Likeness and ADMET Prediction

The drug-likeness properties were calculated using Lipinski’s rule of five [22]. According to this rule, the active compound should have no more than one violation. Lipinski’s rules are the following:

- no more than 5 H bond donors (OH, NH, and SH);
- no more than 10 H bond acceptors (N, O, and S atoms);
- molecular weight below 500 Da;
- octanol-water partition coefficient (log P) below 5.

The SwissADME server (Switzerland) [23] was used for the evaluation of the drug-likeness properties. The SwissADME [23] and pkCSM (Australia) [24] servers were used to predict the pharmacokinetic parameters of absorption, distribution, metabolism, excretion, and toxicity (ADMET).

### 2.4. Molecular Dynamics Simulations

Molecular dynamics computations were performed in YASARA (Austria) [25,26]. All the molecular dynamics simulations were run with the AMBER14 forcefield [27] using a multiple time step of 1.25 fs for intramolecular and 2.5 fs for intermolecular forces. Simulations were performed in cuboid cells with dimensions of 94 × 89 × 72 Å, and counter-ions (56 Cl^−^ and 40 Na^+^) were added to a final concentration of 0.9% NaCl. In total, each simulation contained approximately 62,000 atoms. An 8 Å cutoff was taken for Lennard-Jones forces and the direct space portion of the electrostatic forces, which were calculated using the Particle Mesh Ewald method [28] with a grid spacing <1 Å, 4th order B-splines, and a tolerance of 10^−4^ for the direct space sum. The simulated annealing minimizations started at 298 K, and the velocities were scaled down by 0.9 every ten steps for a total time of 5 ps. After annealing, simulations were run at 298 K. The temperature was adjusted using a Berendsen thermostat [29] based on the time-averaged temperature, i.e., to minimize the impact of temperature control, the velocities were rescaled only about every 100 simulation steps, whenever the average of the last 100 measured temperatures converged. Substrate parameterization was performed with the AM1BCC method [30,31]. All the simulations were run for approximately 150 ns.

## 3. Results and Discussion

### 3.1. Docking

During the virtual screening of the 1893 terpenes used, 20 were selected based on their high scores (Table 1 and Table 2). Ranking these 20 results according to their combined PLP.Fitness (GOLD), InstaDock binding free energy, and VINA scores yielded nine candidates for further study. They were gniditrin, mulberrofuran G, cochlearine A, ingenol dibenzoate, mezerein, pawhuskin B, isogemichalcone C, 3-cinnamyl-4-oxoretinoic acid, and DTXSID501019279. All of these are either among the 3 best candidates in one of the categories, or else among the best 10 in every category.

### 3.2. In Silico Drug-Likeness and ADMET Prediction

According to Lipinski’s rule of five, eight of the selected nine terpenes have one violation, mainly a molecular weight >500. Dasabuvir has 0 violations, while sofosbuvir has 2, including the molecular mass. The in silico drug-likeness and pharmacokinetic properties are presented in Table 3.

In terms of water solubility, sofosbuvir is soluble and dasabuvir is moderately soluble. Among the selected terpenes, most are poorly soluble, but gniditrin, mezerein, ingenol dibenzoate, and pawhuskin B are moderately soluble. Apart from pawhuskin B and ingenol dibenzoate, the gastrointestinal absorption of the studied drugs and terpenes is low. The bioavailability score of terpenes is the same as that for dasabuvir (Table 3).

AMES toxicity and hepatotoxicity are absent from almost all of the selected terpenes (Table 4), except for DTXSID501019279, which demonstrates AMES toxicity and 3-cinnamyl-4-oxoretinoic acid, which exhibits hepatoxicity. The predicted toxicity of these two terpenes may not be disqualifying for their therapeutic use, since both tested drugs also fail at least one of these tests: dasabuvir also presents AMES toxicity, and several reports describe the hepatotoxicity of sofosbuvir [32,33,34]. The hepatotoxicity of dasabuvir was also described in a patient with kidney transplantation [35]. In oral rat acute toxicity, the LD50 concentrations are similar in the drugs and selected terpenes. In the case of oral rat chronic toxicity, the values are generally somewhat higher in the terpenes than sofosbuvir and, especially, dasabuvir. This suggests that these terpenes may be less toxic with prolonged use.

In our study, the in silico activity of a large number of terpenes against HCV NS5B polymerase was tested. In other in silico papers, much smaller amounts of compounds against NS5B were studied, e.g., Ejeh et al. [36] tested 69 molecules, and Rehman et. al. [37] tested 12 compounds in *Taraxacum officinale*. Both articles found substances with better docking properties than sofosbuvir. The authors of the former study also emphasize the lower toxicity of the selected compounds compared to sofosbuvir. Interestingly, oleanolic acid and ursolic acid have been described as good NS5B RdRp inhibitors [14]. In our virtual screening, the PLP.Fitness scores of these two molecules were 49.9716 and 45.2942, respectively. This means that both acids have lower binding potential to NS5B than our nine selected terpenes and studied drugs.

### 3.3. Analysis of the Initial Docking Poses Obtained in the Virtual Screening of the Terpene Collection

**Figure 1:**
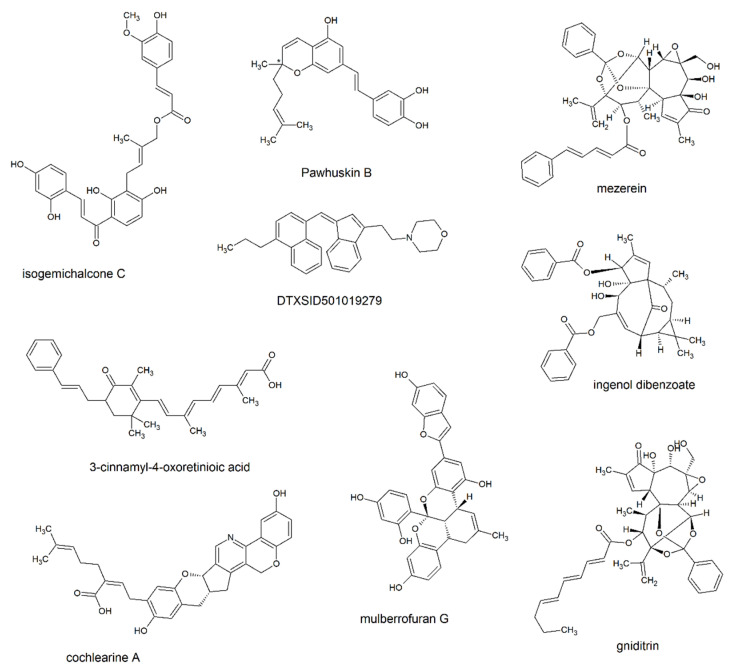
Structures of the selected terpenes. The chiral carbon with unknown stereochemistry in pawhuskin B is highlighted with an asterisk.

**Figure 2:**
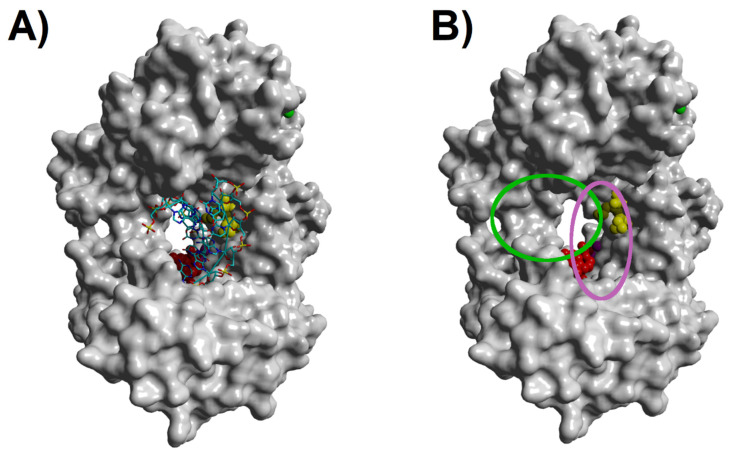
Molecular surface of NS5B RNA polymerase and regions where selected terpenes or proven inhibitors bind. (**A**) RNA-bound NS5B RNA polymerase (PDB: 4WTF) bound to sofosbuvir (red, space-filling model), superposed with HCV-796 (yellow space-filling model). In (**B**), the RNA molecules have been removed to facilitate visualization of the terpene binding regions. Green oval: mezerein/gniditrin binding region; purple oval: binding region of *S*-pawhuskin, isogemichalcone C, and 3-cinnamyl-4-oxo-retinoic acid. Other terpenes bind either to the sofosbuvir or the HCV-796 binding regions.

The nine terpenes selected in the screening stage have a large variety of structures (Figure 1), and they always contain a rigid core (composed of fused rings or highly conjugated systems) and some flexible appendages. The NS5B RNA polymerase active site cavity, in turn, is large enough to accommodate the template RNA chain, the first few nucleotides of the nascent RNA chain, and the nucleotide that must be incorporated in the polymerase reaction (Figure 2A). A comparison of the docking positions of the terpenes reveals that they are predicted to bind to different positions on this surface (Figure 2B). R-pawhuskin and cochlearine A bind at the position occupied by the proven inhibitor HCV-796. Ingenol benzoate and DTXSID501019279 are predicted to bind close to the active site, around the position where the substrate-analog inhibitor sofosbuvir binds. S-Pawhuskin, isogemichalcone C, and 3-cinnamyl-4-oxo-retinoic acid adopt extended conformations that encompass the binding sites of both HCV-796 and sofosbuvir. Mezerein and gniditrin, in contrast, both occupy a region away from the known inhibitor binding sites, with their rigid bridged bicycle cores filling the region where the nascent RNA chain should grow, and their flexible chains extending away from the center of the cavity. Mulberrofuran G, which is the most rigid of these terpenes, occupies a position where its fused cyclic core lies in the region that would be occupied by the ribose sugars of the nascent RNA chain, and its benzofuran-6-ol substituent is pointing towards the active site and barely overlaps with the site where sofosbuvir binds.

Due to the need to sample a very large conformational space, which entails the evaluation of millions of docking poses for each compound, molecular docking relies on quickly computed scoring functions, which have to be approximate and necessarily miss crucial energetic contributions that are hard to predict. For these reasons, molecular docking is remarkably prone to yielding false positives, and its predictions must always be checked, either experimentally or through more detailed computational methods that take into account all the energetic factors, solvation effects, and dynamic fluctuations of the target protein. We, therefore, performed 150 ns-long molecular dynamics simulations of each of the nine protein––terpene complexes predicted by VINA. Analysis of these simulations showed that although the identified terpenes generally remained in the vicinity of the docking site, considerable variation in the stability of the interactions exists, and most of them did not yield stable binding poses, as will be detailed in the following section.

### 3.4. Binding Mode Stability Using Molecular Dynamics

**Figure 3:**
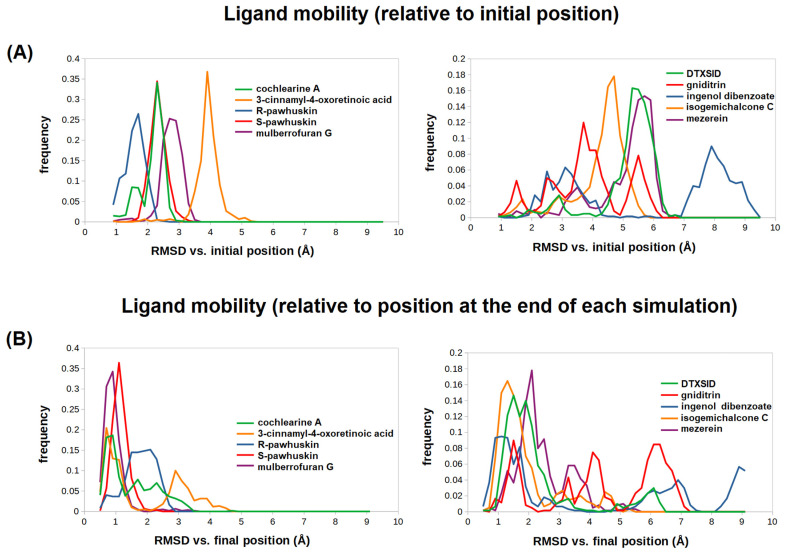
RMSD histograms of the ligands in each complex, measured relative to (**A**) the initial structure or (**B**) the final structure obtained in the 150 ns simulations. In each case, the ten ligands have been plotted in two panels to prevent overcrowding.

Analysis of the trajectories of each individual protein–terpene complex is a complex endeavor due to the large number of coordinates of the molecular system, which creates obvious difficulties for information extraction. Many of those coordinates do not evolve independently but are instead highly correlated at the level of individual functional groups, sidechains, and backbone atoms, and, therefore, more compact information measures that summarize the changes in the position, orientation, or conformation of a ligand (or protein) relative to a given reference structure can be defined, at the cost of losing some detail (of varying importance). One such measure is the root-mean-squared-deviation (RMSD), which is computed by superposing the structures obtained at two different points of the trajectory, measuring the displacement of each atom, averaging the squares of those displacements, and taking the square root of the resulting quantity. If the structure superposition neglects the protein atoms and is instead performed considering only the positions of the ligand atoms, the RMSD will only depend on the flexibility of the ligand and yield no information regarding the stability of the docking position. However, if the superposition takes into account all the atoms in the protein–ligand complex, RMSD allows us to compute the relative magnitudes of the conformational changes of each component of a complex, regardless of whether they are due to translation, rotation, or conformational flexibility. The unidimensional nature of RMSD (which is a single number) does not, however, allow one to distinguish whether its variations are due to rotation, translation, or flexibility. It is especially notable that when comparing the RMSDs of a rigid molecule (which depend only on translation/rotation) and those of a comparable molecule with flexible sidechains, the more flexible molecule may show a larger RMSD (due to conformational variation around some of its internal bonds) despite remaining translationally and rotationally more invariant than the rigid molecule. Despite these limitations, the analysis of the evolution of the RMSD (vs. a reference conformation) along a trajectory may, if properly performed, allow the quick discrimination between ligands that remain tightly bound in their original docking position from those that tend to wander around (or away from) the docking site. An analysis of the RMSD histograms of each ligand in the respective complex, computed vs. the initial (Figure 3A) simulation snapshot, shows that cochlearine A and the two stereoisomers of pawhuskin B remain within 2.5 Å of their initial geometry more than 85% of the simulation time, whereas isogemichalcone C, mezerein, 3-cinnamyl-4-oxo-retinoic acid, and DTXSID501019279 spend more than 90% of the simulation time 2.5 Å away from their original binding pose, and ingenol dibenzoate spends most of the simulation time more than 6 Å away from its starting pose.

A comparison of the RMSD histograms is, however, not enough to confidently classify the ligands into good and poor binders. For example, if two molecules both contain a moiety that remains immobile and tightly bound to the active site and a “tail” that protrudes away from the active site without any influence on the binding affinity, the ligand with the most flexible (and/or longest) tail will necessarily have a larger RMSD than the other, simply due to the larger conformational freedom of its tail. In this case, a deeper analysis where the evolution of key protein–ligand distances is studied or the RMSD is measured against a different reference snapshot may show that removing the ligand with a relatively large RMSD from further consideration would be a poor decision.

We have, therefore, also measured the ligand RMSD relative to the geometry reached at the end of the simulation (Figure 3B, and Table 5). The very high RMSD values of gniditrin are herein confirmed to be independent of the reference snapshot and are, therefore, due to its large mobility in the active site, showing that it does not achieve strong binding to the active site cavity. Mezerein and ingenol dibenzoate take a long time (Table 5) to attain conformations close to their final binding poses, which are moreover quite far from the ones predicted by docking. These observations suggest that the final poses are not stable, and, indeed, a careful analysis of the evolution of the distances between the ligand and active site features (Supporting Information) shows that ingenol dibenzoate will, at most, bind in a position where it will be unable to interfere with the outcome of the reaction and that mezerein never reaches a tight binding position at all. The relatively low RMSD measured vs. the final position in >50% of the trajectory is due to its rigidity, and it actually reflects sizeable fluctuations in the orientation and position of the center of mass of mezerein.

Mulberrofuran G is hereby shown to spend 99% of the simulation close to the position observed at the end. Since the only flexible portion of mulberrofuran G can mostly rotate around a single bond and, therefore, contributes little to the overall RMSD change, the change in the RMSD observed for this ligand is almost completely due to an initial small translation/rotation of the ligand within the active site pocket, followed by relative immobility. This pose overlaps both the sofosbuvir binding site and the binding site of the first two to three nucleotides of the nascent RNA chain, which (in combination with its extreme stability) strongly implies that mulberrofuran G should be a good competitive inhibitor. Isogemichalcone C (which contains two rotatable C-C bonds in the middle of its extensive structure) and DTXSID501019279 (which contains a propyl and an ethylmorpholino substituents) are much more flexible than mulberrofuran G and cannot achieve low-RMSD poses unless the conformational freedom around those bonds is considerably restricted. Still, they quickly adopt stable binding modes for over 80% of the simulation time, which shows that the molecules must have been efficiently locked into place. Plotting the RMSD (relative to the final complex structure) vs. the simulation time (Supporting Information) reveals that upon 20 ns of simulation time, isogemichalcone C and DTXID501019279 adopt a binding mode lying within 2 Å of the final simulation geometry. After 66 ns, 3 cinnamyl-4-oxo-retinoic acid (which contains a flexible cinnamyl substituent bound to a rigid retinoic acid moiety) also becomes locked into a conformation barely 1 Å away from that observed at the end of the simulation. This observation suggests that prior to 66 ns, the cinnamyl moiety was free to rotate, and it then became tightly bound to the binding site. 

The interpretations of the observed evolution of the RMSD in the preceding paragraph are confirmed by further inspection of the trajectories and analyses of the evolution of specific ligand-active site distances throughout the simulations (Supplementary materials). It can be seen that DTXSID501019279 quickly converts from a bent, U-shaped, conformation into a more extended conformation, with the morpholine end relatively close to the initial site but with large displacements of the propylnaphtalene end into a pocket around the position occupied by the second deoxyribose of the 3′ end of the nascent RNA chain in PDB:4WTF. After this movement, which is complete after 20 ns, the ligand mobility is strongly reduced. The rigid core of isogemichalcone C, in turn, remains practically immobile at the binding site of HCV-796 throughout the simulation, and practically all of the RMSD change occurs as a consequence of the conformational flexibility of its other half, which explores the surroundings for approximately 60 ns before becoming locked in place. 3-cinnamyl-4-oxoretinoic acid, like DTXSID501019279, initially binds in a V-shaped conformation, but it eventually reaches a linear, very stable conformation where even its flexible portion is firmly locked in place (ligand RMSD 0.95 ± 0.39 Å vs. the 75 ns snapshot in the 60.5–150 ns interval). This extension process is slower than that of DTXSID501019279, but the good stability of this binding mode and its overlap with both inhibitor binding sites and the RNA-binding interface strongly support its potential as a promising inhibitor too.

## 4. Conclusions

The results obtained indicate that some terpenes exhibit strong, stable binding to HCV RNA-dependent RNA polymerase NS5B. Molecular dynamics simulations allowed us to discard gniditrin, ingenol dibenzoate, and mezerein from further consideration due to the low stability of their positions and the high variability in the distances between them and important active site features. In addition, the simulations suggest that both stereoisomers of pawhuskin B, cochlearine A, and mulberrofuran G are very promising candidates for potential use as HCV drugs or compounds supporting the treatment of this infection. Isogemichalcone C, cochlearine A, DTXSID501019279, and 3-cinnamyl-4-oxoretinoic acid also afford stable binding interactions, though only after a short lag caused by the relatively poor quality of their initial docking poses. The very high stability of the binding modes that they eventually adopted argues in favor of their potential application as future inhibitors. A combination of all of these results with the ADMET predictions suggests that the best candidates will be the two stereoisomers of pawhuskin B, which have extremely tight binding, good gastrointestinal absorption, and no predicted hepatoxicity.

## Figures and Tables

**Table 1:** Chemical structures and molecular weights of 20 selected terpenes and 2 drugs, sofosbuvir and dasabuvir.

PubChem CID	Compound Name	Chemical Structure	Molecular Weight [g/mol]
45375808	Sofosbuvir	C_22_H_29_FN_3_O_9_P	529.5
56640146	Dasabuvir	C_26_H_27_N_3_O_5_S	493.6
5281368	Gniditrin	C_37_H_42_O_10_	646.7
196583	Mulberrofuran G	C_34_H_26_O_8_	562.6
122376979	Cochlearine A	C_31_H_29_NO_6_	511.6
44369392	Ingenol Dibenzoate	C_34_H_36_O_7_	556.6
5281382	Mezerein	C_38_H_38_O_10_	654.7
11199792	Pawhuskin B	C_24_H_26_O_4_	378.5
5287705	ALRT 1550	C_23_H_32_O_2_	340.5
21589718	Hydrangenoside E	C_29_H_40_O_12_	580.6
53963605	DTXSID70708006	C_23_H_32_O_2_	340.5
46831971	Expansol B	C_29_H_36_O_5_	464.6
10143276	Isogemichalcone C	C_30_H_28_O_9_	532.5
72950872	DTXSID501019279	C_29_H_31_NO	409.6
122177658	Peniciaculin B	C_30_H_44_O_6_	500.7
10320495	3-Cinnamyl-4-Oxoretinoic Acid	C_29_H_34_O_3_	430.6
11394888	Pawhuskin C	C_24_H_28_O_4_	380.5
5281391	Phorbol Caprate	C_35_H_52_O_8_	600.8
24766094	Perrottetinene	C_24_H_28_O_2_	348.5
5471965	Presqualene Alcohol	C_30_H_50_O	426.7
6449829	AC-5-1	C_25_H_30_O_5_	410.5
10027720	Mispyric acid	C_30_H_46_O_4_	470.7

**Table 2:** Results of docking scores for 20 selected terpenes and 2 anti-HCV drugs. Scores more favorable than those of sofosbuvir or dasabuvir are highlighted in bold.

Compound Name	VINA Score	GOLD PLP.Fitness	GOLD RMSD	INSTA Dock “Binding Free Energy” (kcal/mol)	Ligand Efficiency (kcal/mol/non-H Atom)
**Sofosbuvir**	9.8	58.9498	2.1–9.4	−8.1	0.162
**Dasabuvir**	9.4	62.5419	1.5–11	−9.0	0.1957
**Gniditrin**	8.5	**92.6287**	1.5–9.4	**−9.2**	0.1415
**Mulberrofuran G**	**12.0**	**91.4167**	1.5–9.1	**−9.6**	0.1778
**Cochlearine A**	**9.6**	**88.2803**	1.5–3.7	**−9.8**	0.1885
**Ingenol Dibenzoate**	**10.0**	**87.2843**	1.5–8.9	**−10.1**	0.1836
**Mezerein**	8.5	**86.8922**	1.2–9.1	**−9.9**	0.1547
**Pawhuskin B**	8.9	**90.9802**	1.5–4.8	**−8.9**	0.2171
ALRT 1550	8.1	**90.9274**	1.5–7.5	−7.5	0.2027
Hydrangenoside E	8.6	**89.5570**	1.5–4.5	**−8.2**	0.1302
DTXSID70708006	8.1	**89.1282**	1.5–8.4	−7.5	0.2027
Expansol B	8.7	**89.0118**	1.5–5	**−8.8**	0.1692
**Isogemichalcone C**	**11.0**	**88.5244**	1.5–6.8	−7.8	0.13
**DTXSID501019279**	9.1	**87.8244**	1.6–9.1	**−8.9**	0.2119
Peniciaculin B	8.0	**87.5688**	2.4–11.9	−7.5	0.1271
**3-Cinnamyl-4-Oxoretinoic Acid**	**10.0**	**87.0663**	1.5–3.7	**−8.4**	0.1826
Pawhuskin C	8.9	**86.4053**	1.5–4.3	**−8.9**	0.2171
Phorbol Caprate	6.8	**86.0402**	1.5–10.5	−7.7	0.1185
Perrottetinene	**9.5**	**85.9861**	1.5–8.9	**−8.3**	0.2371
Presqualene Alcohol	6.4	**85.5873**	1.5–11.6	−7.1	0.1392
AC-5–1	8.9	**85.3062**	1.4–5	−7.5	0.1562
Mispyric acid	9.1	**84.8975**	1.7–12.8	−7.9	0.1463

**Table 3:** Results of in silico drug-likeness and pharmacokinetic properties of selected 5 terpenes and 2 anti-HCV drugs.

Compound Name	#H Bond Acceptors	#H Bond Donors	Lipophilicity (Log P)	Water Solubility	Gastrointestinal Absorption	Lipinski #Violations	Bioavailability Score
Sofosbuvir	11	3	1.44	Soluble	Low	2	0.17
Dasabuvir	5	2	3.80	Moderately soluble	Low	0	0.55
3-Cinnamyl-4-Oxoretinoic_Acid	3	1	5.84	Poorly soluble	Low	1	0.55
Cochlearine A	7	3	4.69	Poorly soluble	Low	1	0.56
DTXSID501019279	2	0	5.89	Poorly soluble	Low	1	0.55
Gniditrin	10	3	3.74	Moderately soluble	Low	1	0.55
Ingenol Dibenzoate	7	2	4.14	Moderately soluble	High	1	0.55
Isogemichalcone_C	9	5	4.26	Poorly soluble	Low	1	0.55
Mezerein	10	3	3.15	Moderately soluble	Low	1	0.55
Mulberrofuran G	8	5	4.90	Poorly soluble	Low	1	0.55
Pawhuskin_B	4	3	4.77	Moderately soluble	High	0	0.55

**Table 4:** Results of toxic properties of selected terpenes and 2 anti-HCV drugs.

Molecule	AMES Toxicity	Oral Rat Acute Toxicity (LD50) [mol/kg]	Oral Rat Chronic Toxicity (LOAEL) [log mg/kg_bw/day]	Hepatotoxicity	Skin Sensitisation
Sofosbuvir	No	2.618	2.402	Yes	No
Dasabuvir	Yes	2.944	1.796	Yes	No
Pawhuskin_B	No	2.375	1.02	No	No
DTXSID501019279	Yes	2.388	0.051	No	No
3-Cinnamyl-4-Oxoretinoic_Acid	No	2.1	2.392	Yes	No
Cochlearine A	No	2.871	2.015	No	No
Gniditrin	No	3.277	2.380	No	No
Ingenol Dibenzoate	No	2.383	2.047	No	No
Isogemichalcone_C	No	2.385	3.565	No	No
Mezerein	No	2.668	3.131	No	No
Mulberrofuran G	No	2.549	3.097	No	No

**Table 5:** Summary of the binding stability characteristics of each complex.

	Time Ligand Spent within 2.5 Å of Its Initial Position	Time Ligand Spent within 2.5 Å of Its Final Position	When Does It Reach a Relatively Stable Conformation within 2 Å RMSD of the Final Geometry?	Ligand RMSD in the Complex at the End of the Simulation
DTXSID501019279	4%	81%	25 ns	5 Å
cochlearine A	96%	89%	50 ns	2.1 Å
gniditrin	17%	23%	115.5 ns	5.9 Å
ingenol dibenzoate	11%	53%	72.25 ns	7.9 Å
mezerein	4%	68%	56 ns	5.5 Å
mulberrofuran G	28%	99%	3.5 ns	2.6 Å
isogemichalcone C	7%	81%	31 ns	4.6 Å
(R)-pawhuskin	100%	98%	Immediately	1.8 Å
(S)-pawhuskin	86%	100%	0.25 ns	2.1 Å
3-cinnamyl-4-oxoretinoicacid	2%	60%	63.5 ns	3.8 Å

## Data Availability

Please contact T.M.K. and P.J.S. The molecular dynamics snapshots and analysis spreadsheets can be downloaded from Figshare (10.6084/m9.figshare.23214686).

## Supplementary Materials

The following supporting information can be downloaded at https://www.mdpi.com/article/10.3390/pathogens12060842/s1, The file “Supplementary materials” contains a more detailed description of each of the individual trajectories, with supporting graphs of the evolution of key ligand–protein distances as well as graphs showing the evolution of the ligand RMSD along the trajectory.
