# Supplementary material for: Discovery of Terpenes as Novel HCV NS5B Polymerase Inhibitors via Molecular Docking
Source: Pathogens. 2023 Jun 18;12(6):842. doi: 10.3390/pathogens12060842 (PMC10305183; doi:10.3390/pathogens12060842)
Supplement: Supplementary file 1 [file pathogens-12-00842-s001.zip › pathogens-2013194-supplementary.pdf]

Supporting Information for

"Discovery of terpenes as novel HCV NS5B polymerase inhibitors via molecular docking"

Tomasz M. Karpiński [1,*], Marcin Ożarowski [2], Pedro J. Silva [3,4], Mark Stasiewicz [5], Rahat Alam [6], Abdus Samad [6]

[1] Chair and Department of Medical Microbiology, Poznań University of Medical Sciences, Rokietnicka 10, 60-806 Poznań, Poland; tkarpin@ump.edu.pl
[2] Department of Biotechnology, Institute of Natural Fibres and Medicinal Plants – National Research Institute, Wojska Polskiego 71b, 60-630 Poznań, Poland; marcin.ozarowski@iwnirz.pl
[3] FP-I3ID/Fac. de Ciências da Saúde, Universidade Fernando Pessoa, Porto, Portugal; pedros@ufp.edu.pt
[4] UCIBIO@REQUIMTE, BioSIM, Departamento de Biomedicina, Faculdade de Medicina, Universidade do Porto, Porto, Portugal
[5] Research Group of Medical Microbiology, Chair and Department of Medical Microbiology, Poznań University of Medical Sciences, Rokietnicka 10, 60-806 Poznań, Poland; 84682@student.ump.edu.pl
[6] Biological Solution Centre (BioSol Centre), Farmgate, Dhaka-1215, Bangladesh. rahat_160619@just.edu.bd (R.A), kazisamad50@gmail.com (A.S.)

* Correspondence: tkarpin@ump.edu.pl; pedros@ufp.edu.pt

Table of contents

Time evolution of RMSD (vs. initial conformation) 3

Time evolution of RMSD (vs. final conformation) 4

(R)-Pawhuskin B 5

(S)-Pawhuskin B 6

Cochlearine A 7

Mezerein 8

DTXSID501019279 9

Mulberrofuran G 10

Isogemichalcone C 11

Ingenol dibenzoate 12

3-cinnamyl-4-oxoretinoic acid 13

Gniditrin 14

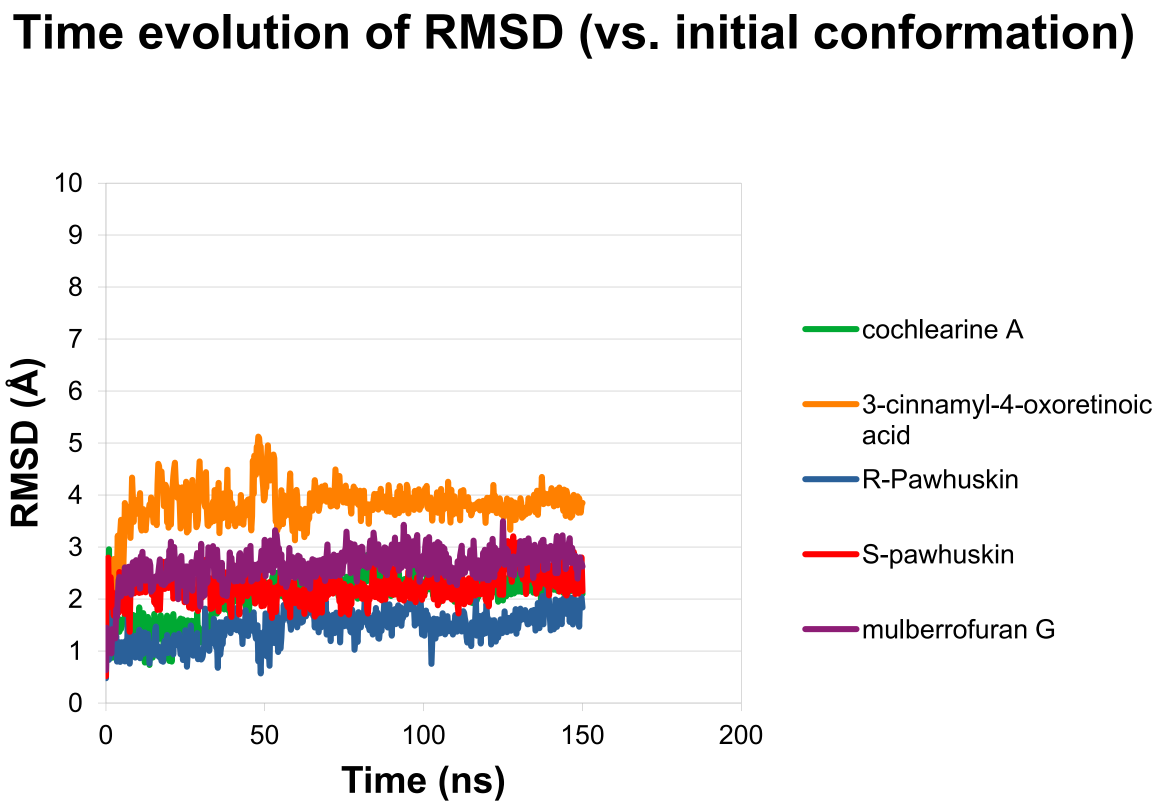

Figure S1: Evolution of RMSD of selected ligands in the respective complexes (vs. initial complex conformation)

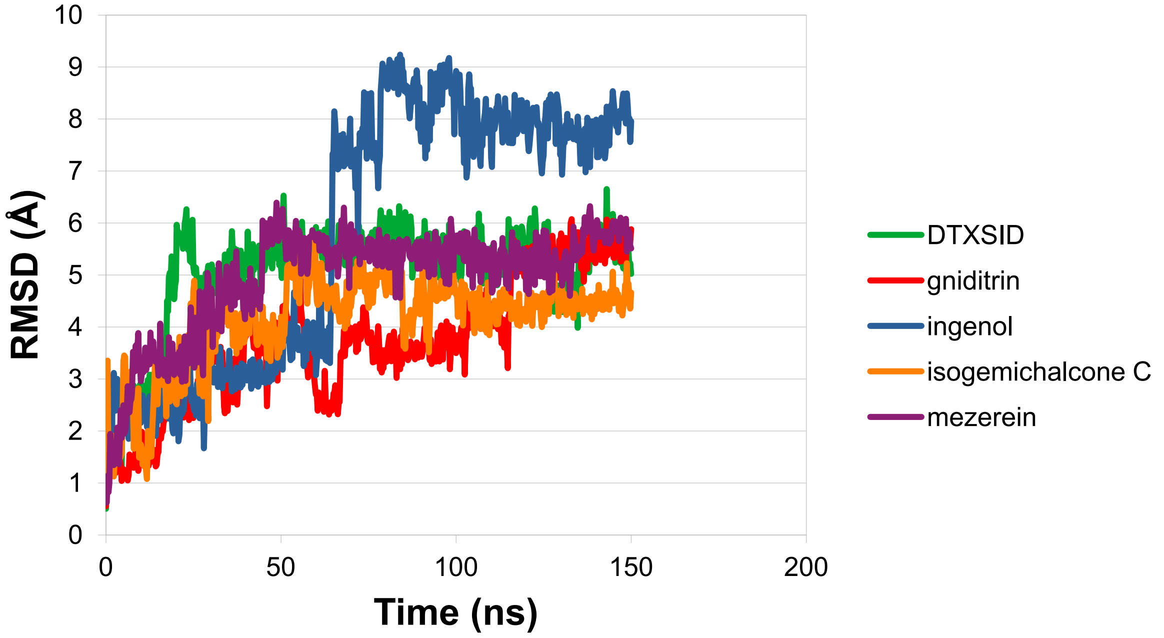

Figure S2: Evolution of RMSD of selected ligands in the respective complexes (vs. initial complex conformation)

Time evolution of RMSD (vs. final conformation)

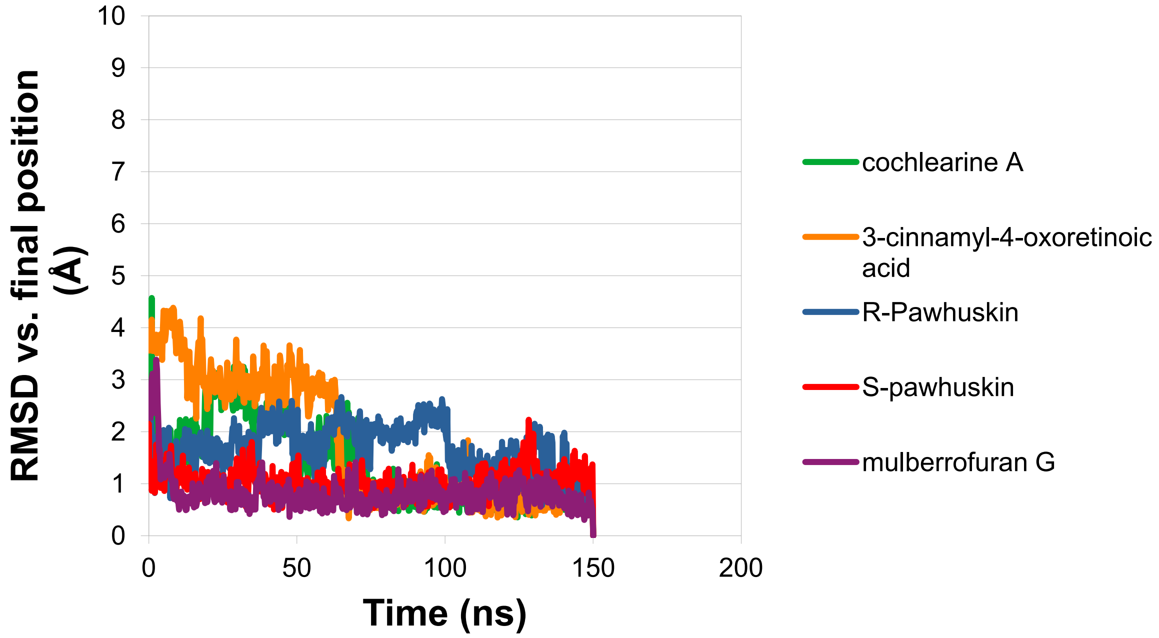

Figure S3: Evolution of RMSD of selected ligands in the respective complexes (vs. final complex conformation)

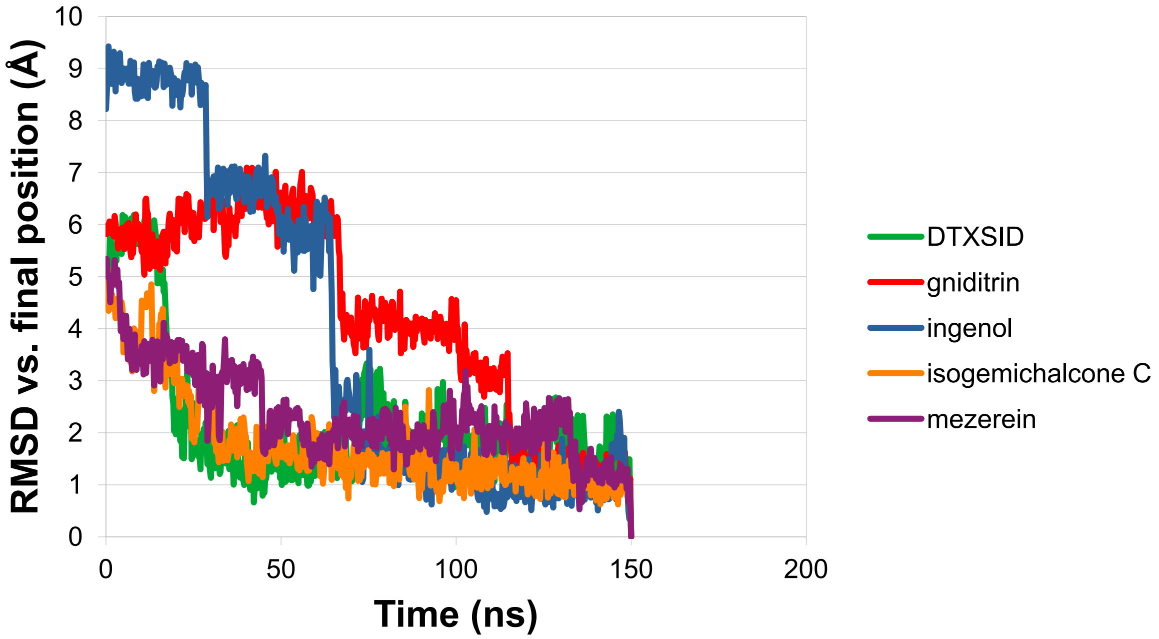

Figure S4: Evolution of RMSD of selected ligands in the respective complexes (vs. final complex conformation)

(R)-Pawhuskin B

The smallest variation between VINA-docking pose and MD simulation was observed for pawhuskin B, with an average root-mean-squared deviation in terpene position of only 1.5 Å relative to the initial pose (measured in the interval 5-150 ns). Most of the molecule has, however a much lower RMSD, since observation of key distances between binding site aminoacid Cα atoms and specific regions of pawhuskin (Figure S1B) show that most of the molecule remains practically immobile throughout the simulation, and only the ligand's geranyl tail changes conformation, due to its position in the open environment where eventually the reaction will take place. The binding position of the geranyl tail can be clearly seen (Figure 2) to partially overlap with the active site region where nucleotides should be adding to the nascent RNA chain, whereas the rest of pawhuskin occupies the site where inhibitor HCV-796 is known to bind, entailing that pawhuskin B is likely to be a good inhibitor.

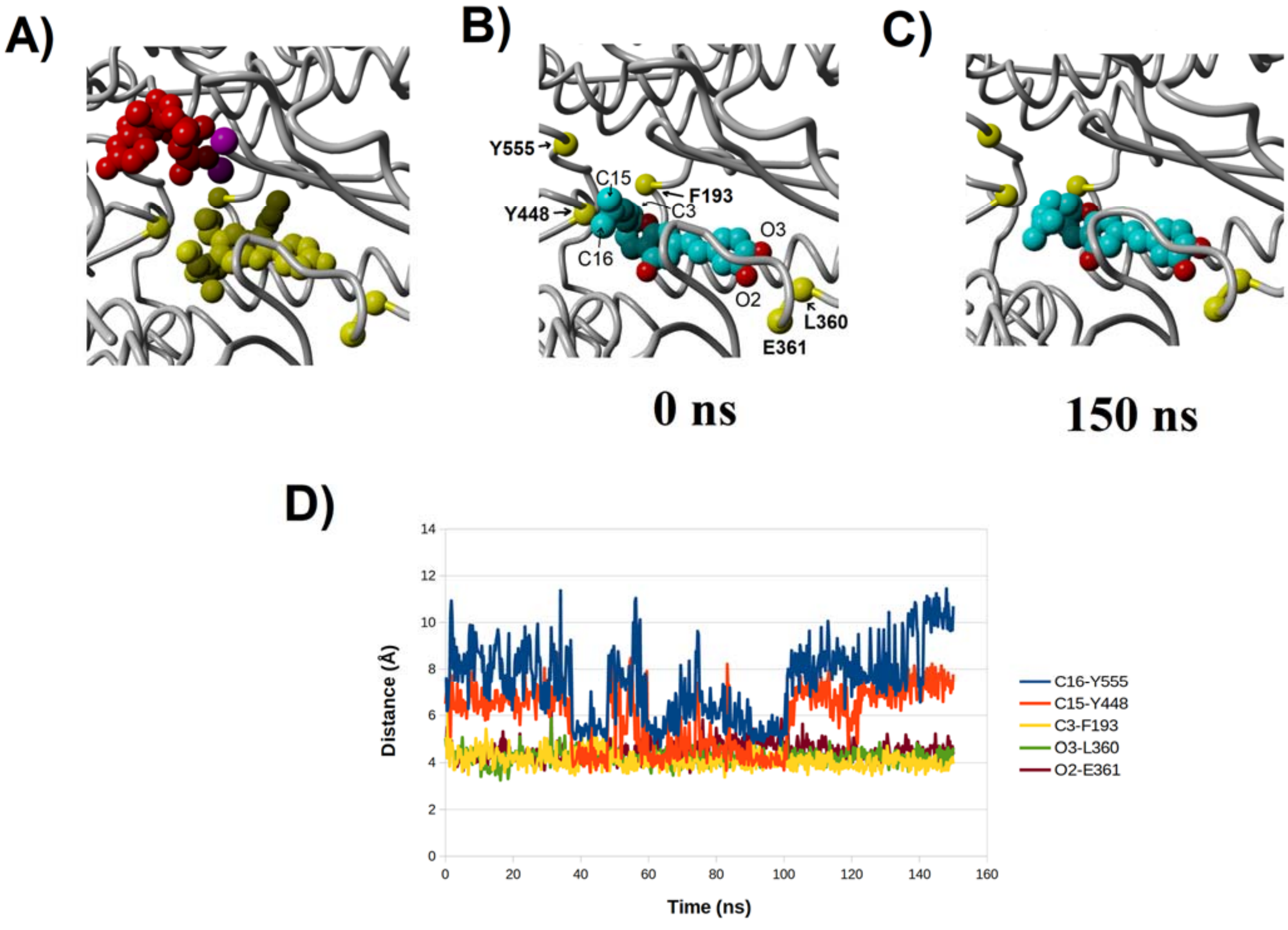

Figure S5: Evolution of the interaction of (*R*)-pawhuskin B with NS5B polymerase. For ease of comparison, the structures shown in panels A, C, and D are depicted after superposing them on each other, so that they have precisely the same orientation. A) polymerase bound to sofosbuvir (red) from PDB:4WTF. The inhibitor HCN-796 (from PDB:3FQK) is shown in yellow; B).Initial docking pose of pawhuskin B (ball-and-sticks model). Several Cα atoms, whose distances to the ligand are depicted in panel D, are shown as yellow balls; C) Final snapshot of the simulation of the ligand-protein complex. ; D) Evolution of key ligand-protein distances along the simulation. Legend refers to the atoms labeled in panels B and C

(S)-Pawhuskin B

Pawhuskin B contains a single stereocenter, whose absolute configuration has not yet been determined. Therefore, we decided to also dock the optical isomer of the Pawhukin B structure studied above. This steroisomer ((S)-pawhuskin B) binds with a good VINA score (8.90), and the molecular dynamics simulation of its complex with the protein shows a well-behaved binding profile, with an average root-mean-squared deviation in terpene position of only 2.2 Å (relative to the initial pose, measured in the interval 5-150 ns). This pose is quickly attained, after less than 10 ns have elapsed: indeed, the average RMSD measured relative to the 25 ns-pose in the 10ns-150 ns interval is as low as 0.91 Å. The binding position can be clearly seen to be approximately "flipped" relative to that of (R)-pawhuskib B (Figure S2): the geranyl portion is now occupying the position where HCV-796 binds, whereas the ring portion fills the region where the nascent RNA chain should form (especially close to Ser 288 and Ser 556).

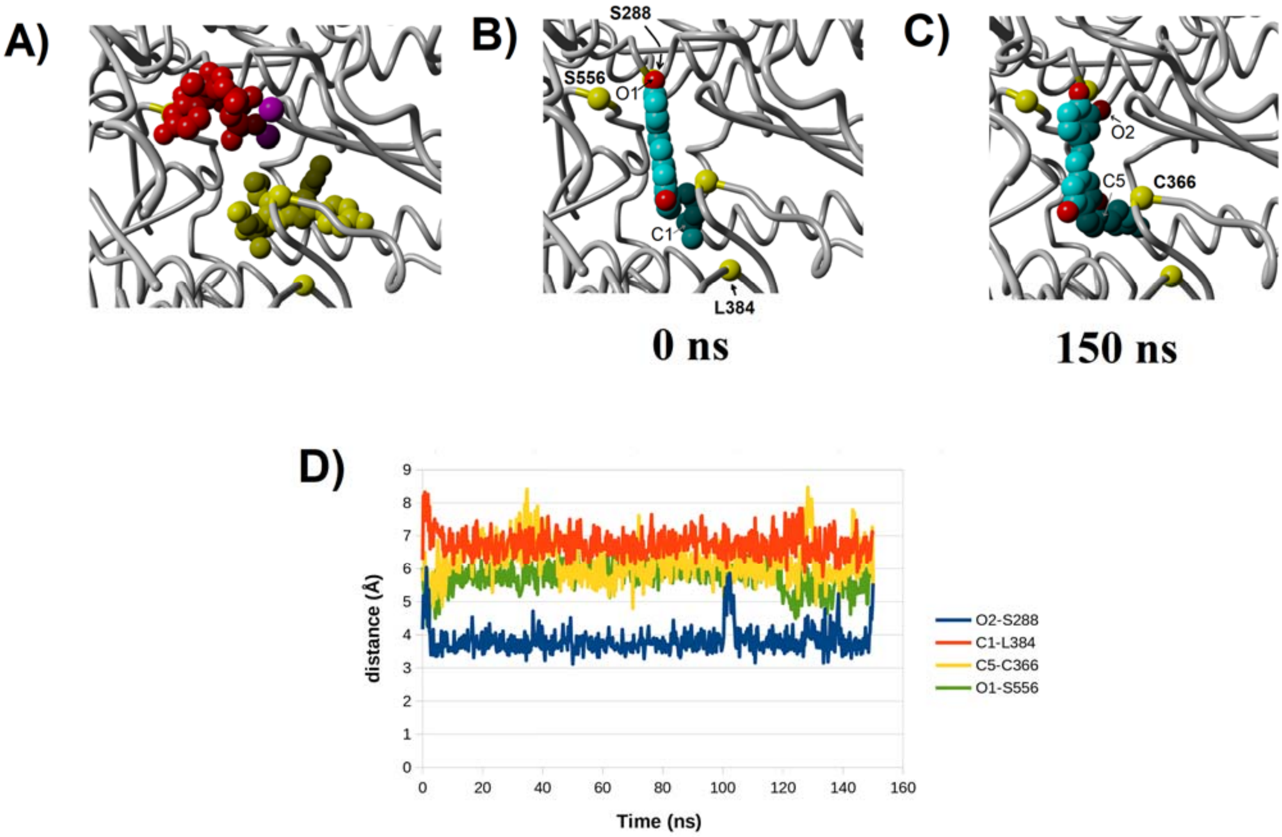

Figure S6: Evolution of the interaction of (*S*)-pawhuskin B with NS5B polymerase. For ease of comparison, the structures shown in panels A, C, and D are depicted after superposing them on each other, so that they have precisely the same orientation. A) polymerase bound to sofosbuvir (red) from PDB:4WTF. The inhibitor HCN-796 (from PDB:3FQK) is shown in yellow; B) Initial docking pose of pawhuskin B (ball-and-sticks model). Several Cα atoms, whose distances to the ligand are depicted in panel D, are shown as yellow balls; C) Final snapshot of the simulation of the ligand-protein complex. D) Evolution of key ligand-protein distances along the simulation. Legend refers to the atoms labeled in panels B and C

Cochlearine A

The next-least-changed docking pose is that of Cochlearine A, which was predicted by VINA to bind at a region overlapping both the site where inhibitor HCV-796 binds and the position of the nascent RNA chain (Figure S3). Throughout the simulation, ligand position has an average RMSD of 2.1 Å relative to the initial pose (measured in the interval 5-150 ns). Similar to what was observed for (R)-pawhuskin B, the geranyl tail present in cochlearine A is placed in an open area of the active site , and is therefore considerably more mobile than the rest of the molecule, which binds to the position occupied by HCV-796 and remains tightly bound and mostly immobile throughout the simulation. Observation of the evolution of key ligand-protein distances show that the geranyl tail progressively loses mobility: at 50 ns, it attains a stable distance towards Ile 447, and then, at around 80 ns, its position relative to both Ile447 and Gly449 becomes fixed (Figure S3). In the stable position, cochlearine is well-place to prevent the synthesis of a new RNA chain, since it blocks part of the groove where this RNA chain should grow.

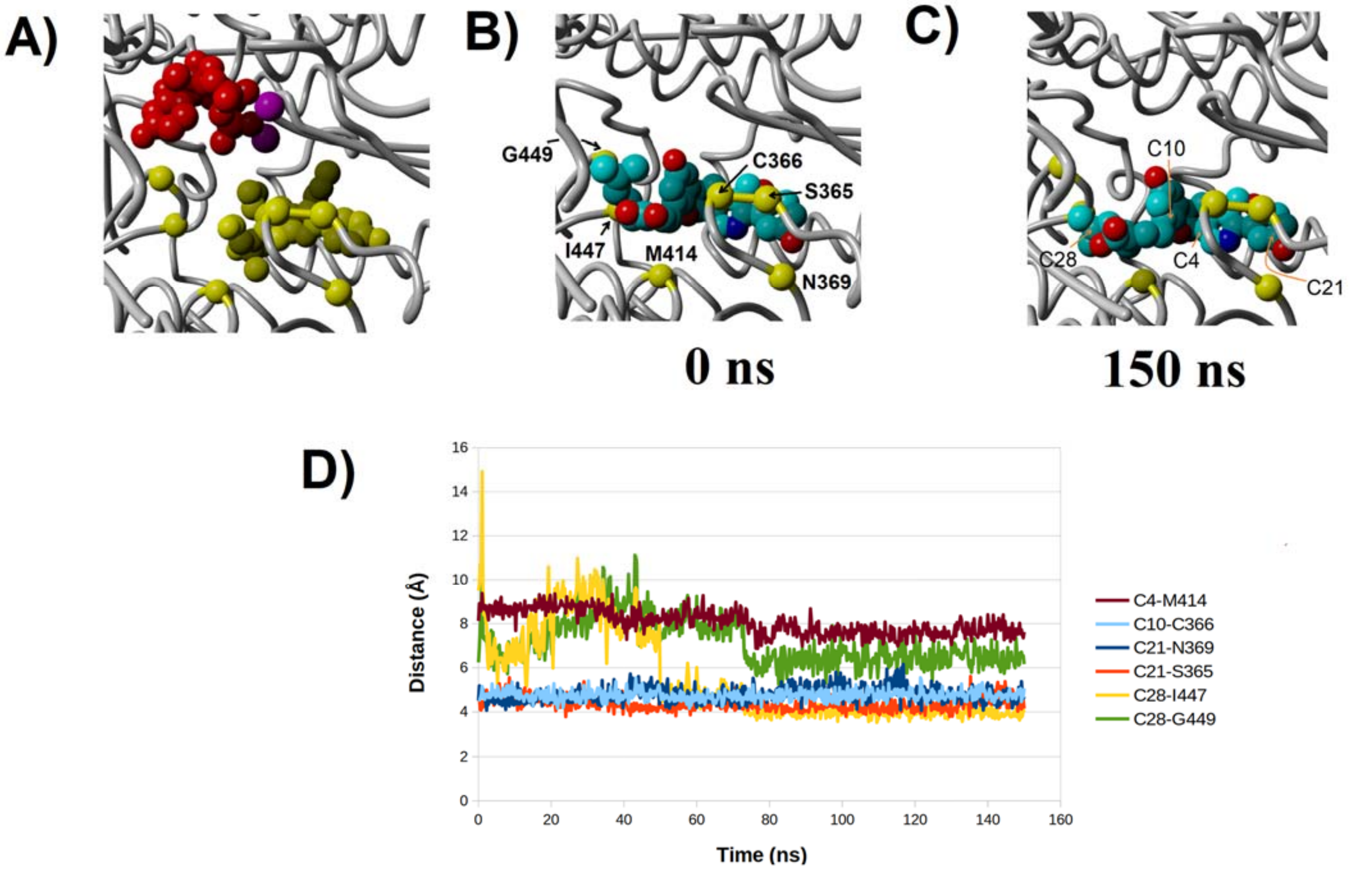

Figure S7: Evolution of the interaction of cochlearine A with NS5B polymerase. For ease of comparison, the structures shown in panels A, B, and C are depicted after superposing them on each other, so that they have precisely the same orientation. A) polymerase bound to sofosbuvir (red) from PDB:4WTF. The inhibitor HCN-796 (from PDB:3FQK) is shown in yellow; B) Initial docking pose of cochlearine A (ball-and-sticks model). Several Cα atoms, whose distances to the ligand are shown in panel D, are depicted as yellow balls; C) Final snapshot of the simulation of the ligand-protein complex. D) Evolution of key ligand-protein distances along the simulation. Legend refers to the atoms labeled in panels B and C.

Mezerein

The initial docking pose of mezerein places it along the position that should be occupied by the nascent RNA chain, with the end of its phenylpentadienoyl moiety close to Glu398 (Figure S4). Very quickly, this region moves away from its initial position, while the whole molecule rotates across its center. The average RMSD of the ligand position (in the 5-150 ns) relative to its initial position is remarkably high, at 5.04 Å, which clearly conveys the magnitude of the overall displacement. The ligand does not attain a proper equilibrium position in the span of the 150 ns-simulation, as can be seen by the evolution of the ligand-protein distances in Figure 5B. This high mobility argues against its possible use as a proper inhibitor of the polymerase activity, since it entails that mezerein's interactions with the active site are too fleeting to be able to compete with the binding of nucleotides.

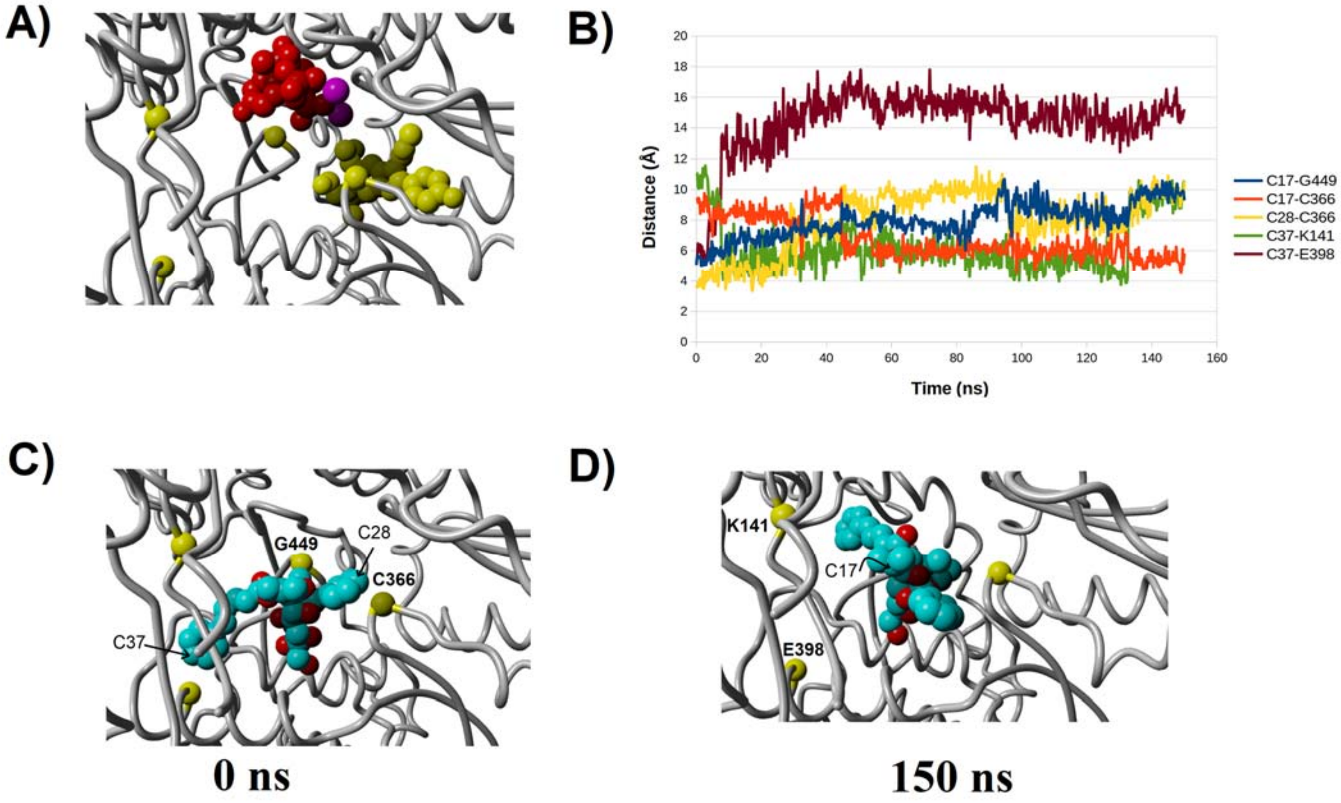

Figure S8: Evolution of the interaction of mezerein with NS5B polymerase. For ease of comparison, the structures shown in panels A, C, and D are depicted after superposing them on each other, so that they have precisely the same orientation. A) polymerase bound to sofosbuvir (red) from PDB:4WTF. The inhibitor HCN-796 (from PDB:3FQK) is shown in yellow; B) Evolution of key ligand-protein distances along the simulation. Legend refers to the atoms labeled in panels C and D; C) Initial docking pose of mezerein (ball-and-sticks model). Several Cα atoms, whose distances to the ligand are shown in panel B, are depicted as yellow balls; D) Final snapshot of the simulation of the ligand-protein complex.

DTXSID501019279

The morpholine-bearing end of DTXSID501019279 docks at the position occupied (in the PDB:4WTF structure) by the 3′-end of the nascent RNA chain, and its propynaphtalene end binds at the position occupied by sofosbuvir in that stucture, so that the initial geometry of DTXSID501019279 at the polymerase binding site is a bent, U-shaped, conformation (Figure S5). As the simulation proceeds, the ligand adopts a more extended conformation, with the morpholine end relatively close to the initial site but with large displacements of the propylnaphtalene end into a pocket defined by Phe193, Arg 200 and Tyr 448. This displacement takes place very quickly, and is basically complete after the first 20 ns have elapsed, and places the naphtalene group in the position occupied by the second deoxyribose of the 3′ end of the nascent RNA chain in PDB:4WTF. From that point on the ligand mostly remains in place apart from a brief movement, between 72 and 85 ns, of the morpholine end of the molecule towards the pocket defined by Asp225, Ser282, Thr287 and Asn291.

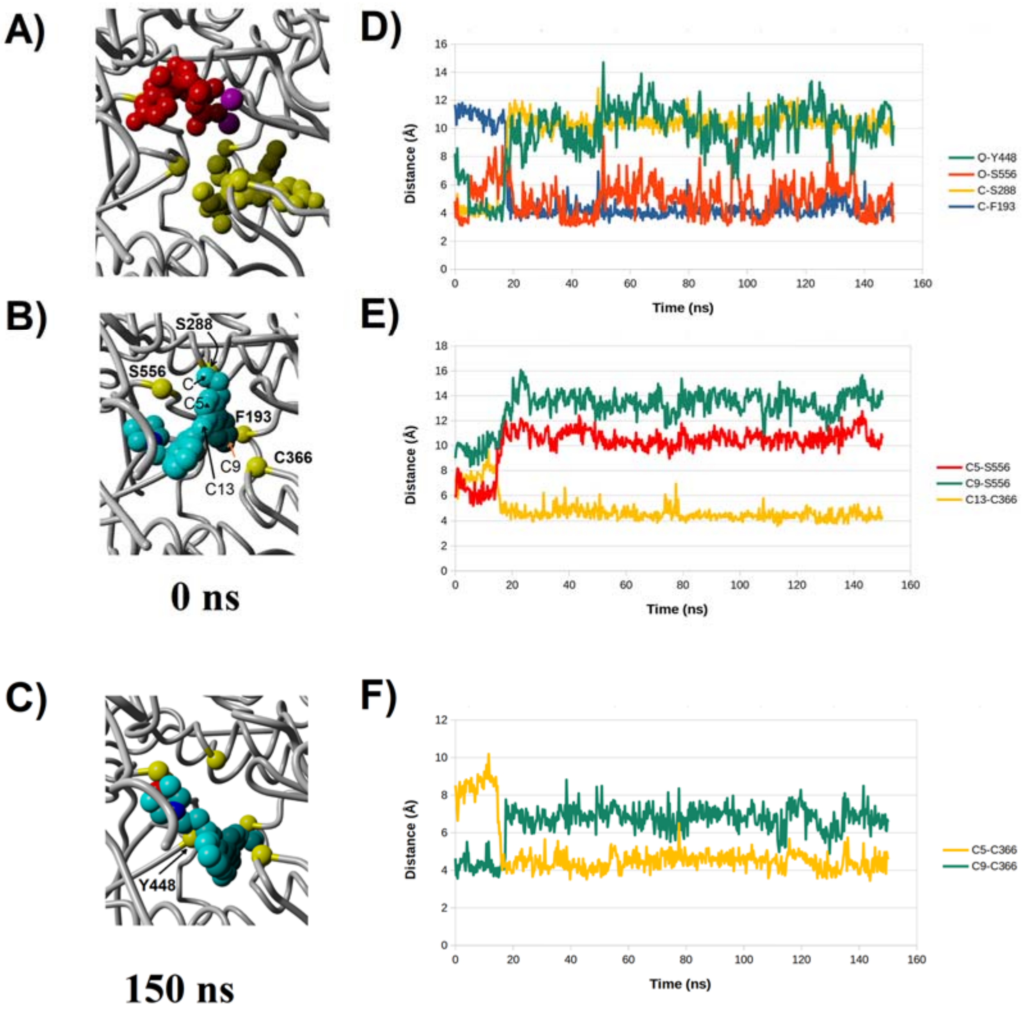

Figure S9: Evolution of the interaction of DTXSID501019279 with NS5B polymerase. For ease of comparison, the structures shown in panels A, B, and C are depicted after superposing them on each other, so that they have precisely the same orientation. A) polymerase bound to sofosbuvir (red) from PDB:4WTF. The inhibitor HCN-796 (from PDB:3FQK) is shown in yellow B) Initial docking pose of DTXSID501019279 (ball-and-sticks model). Several Cα atoms, whose distances to the ligand are shown in panel B, are depicted as yellow balls; C) Final snapshot of the simulation of the ligand-protein complex. D,E,F) Evolution of key ligand-protein distances along the simulation. Legend refers to the atoms labeled in panels B and C.

Mulberrofuran G

Mulberrofuran G, like most of the studied terpenes, binds with one of its ends in the region where the nascent RNA chain grows (Figure S6). It immediately adopts an extremely stable pose (average RMSD of 0.87±0.22 Å in the 5 -150 ns interval, measured against the conformation obtained at 25 ns). This pose overlaps both the sofosbuvir binding site and the binding site of the first 2-3 nucleotides of the nascent RNA chain, which (in combination with its extreme stability) strongly implies that mulberrofuran G should be a good competitive inhibitor.

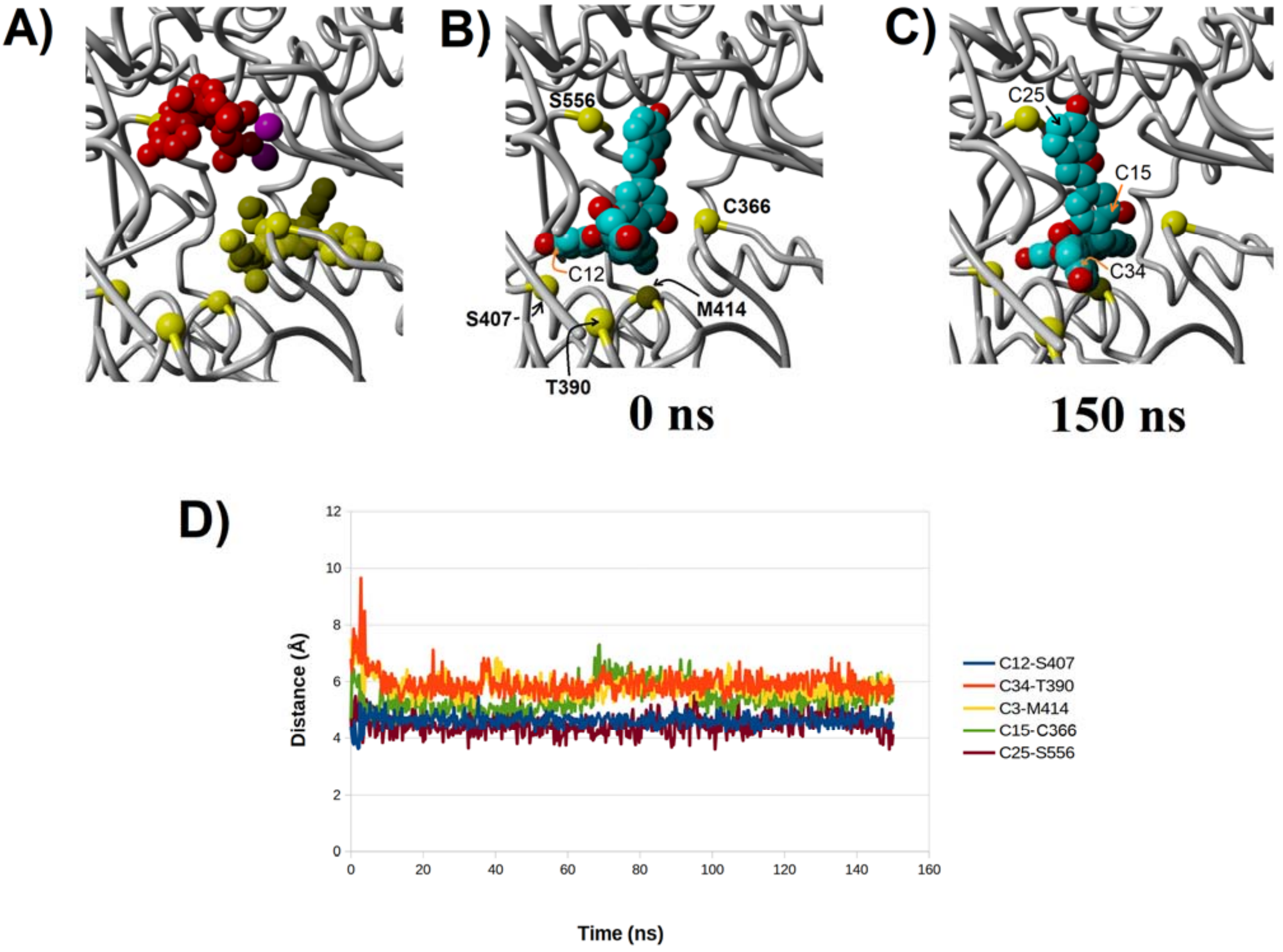

Figure S10: Evolution of the interaction of mulberrofuran G with NS5B polymerase. For ease of comparison, the structures shown in panels A, B, and C are depicted after superposing them on each other, so that they have precisely the same orientation. A) polymerase bound to sofosbuvir (red) from PDB:4WTF. The inhibitor HCN-796 (from PDB:3FQK) is shown in yellow; B) Evolution of key ligand-protein distances along the simulation. Legend refers to the atoms labeled in panels C and D. C) Initial docking pose of mulberrofuran G (ball-and-sticks model). Several Cα atoms, whose distances to the ligand are shown in panel B, are depicted as yellow balls; D) Final snapshot of the simulation of the ligand-protein complex.

Isogemichalcone C

Isogemichalcone C is more mobile in the active site than the previously described pawhuskin B stereoisomers, mulberrofuran G, and cochlearine. Its freedom of movement reflects itself mostly in rotations along the axis defined by the length of isogemichalcone C, rather than on translation movements around (or away from) the active site. Accordingly, the key distances between ligand and Cα of active site residues converge to stable, short, values within 35 ns of the beginning of the simulation (Figure S7), but the ligand displacement RMSDs only become negligible later in the simulation. Then, ligand position RMSD reaches 1.23±0.30 Å in the 55 -150 ns interval, measured against the conformation obtained at 75 ns.

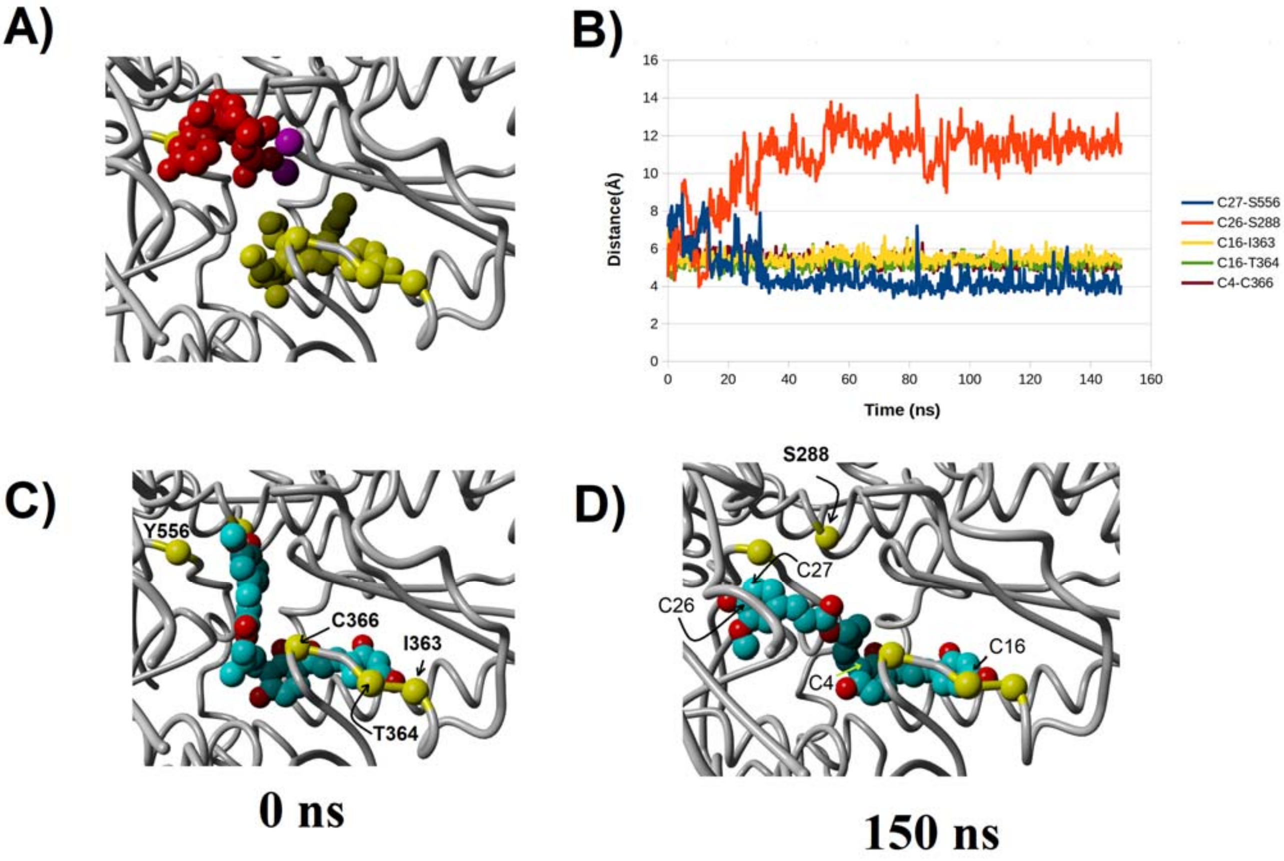

Figure S11: Evolution of the interaction of isogemichalcone C with NS5B polymerase. For ease of comparison, the structures shown in panels A, C, and D are depicted after superposing them on each other, so that they have precisely the same orientation. A) polymerase bound to sofosbuvir (red) from PDB:4WTF. The inhibitor HCN-796 (from PDB:3FQK) is shown in yellow; B) Evolution of key ligand-protein distances along the simulation. Legend refers to the atoms labeled in panels C and D.; C) Initial docking pose of isogemichalcone C(ball-and-sticks model). Several Cα atoms, whose distances to the ligand are shown in panel B, are depicted as yellow balls; D) Final snapshot of the simulation of the ligand-protein complex.

Ingenol dibenzoate

Ingenol dibenzoate also docks, like most other terpenes, at the region encompassing the nascent RNA chain, which at first sight might suggest that it would be a good competitive inhibitor (Figure S8). However, the molecular dynamics simulation shows that this terpene does not attain a stable position within the 150 ns allotted for that: part of the molecule rotates so that its dimethylcyclopropyl moiety quickly leaves the vicinity of Ser226 (where sofosbuvir binds) and moves almost 15 ångstrom away (to the vicinity of Ser366), and even the two dibenzoate groups fail to remain at their initial positions. After 75 ns the ligand does attain a stable conformation (ligand RMSD 1.20±0.43 Å vs. the 150 ns snapshot in the 75-150 ns interval), but the position does not seem conducive to good inhibitory activity because the ligand is barely overlaps the region that is to be occupied by the nascent RNA chain.

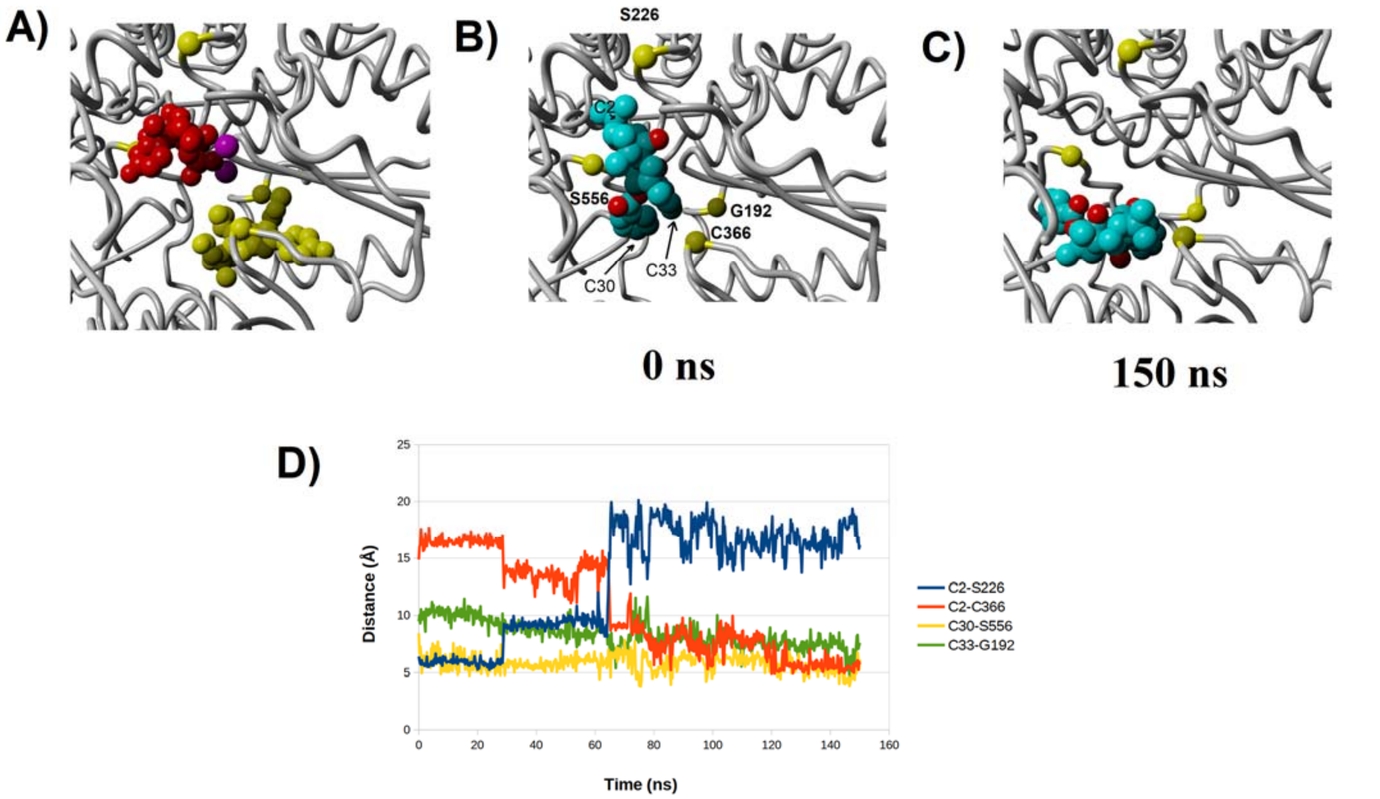

Figure S12: Evolution of the interaction of ingenol dibenzoate with NS5B polymerase. For ease of comparison, the structures shown in panels A, B, and C are depicted after superposing them on each other, so that they have precisely the same orientation. A) polymerase bound to sofosbuvir (red) from PDB:4WTF. The inhibitor HCN-796 (from PDB:3FQK) is shown in yellow; B) Initial docking pose of ingenol dibenzoate (ball-and-sticks model). Several Cα atoms, whose distances to the ligand are shown in panel B, are depicted as yellow balls; C) Final snapshot of the simulation of the ligand-protein complex.; D) Evolution of key ligand-protein distances along the simulation. Legend refers to the atoms labeled in panels B and C.

3-cinnamyl-4-oxoretinoic acid

3-cinnamyl-4-oxoretinoic acid docks with its acid function close to Ser556 and Asn291, in the region where sofosbuvir binds (Figure S9). It initially has a somewhat V-shaped conformation, but it eventually reaches a linear, stable, conformation, where it is fully extended between Ser556 and Ile363. This extension process occurs in two phases: the extension itself is completed within 60 ns, and then the extended molecule moves a few ångstrom towards Phe193, Arg200, and the Leu 360-Cys366 loop. The good stability of this binding mode (ligand RMSD 0.95±0.39 Å vs. the 75 ns snapshot in the 60.5-150 ns interval) suggests this might be a promising inhibitor too.

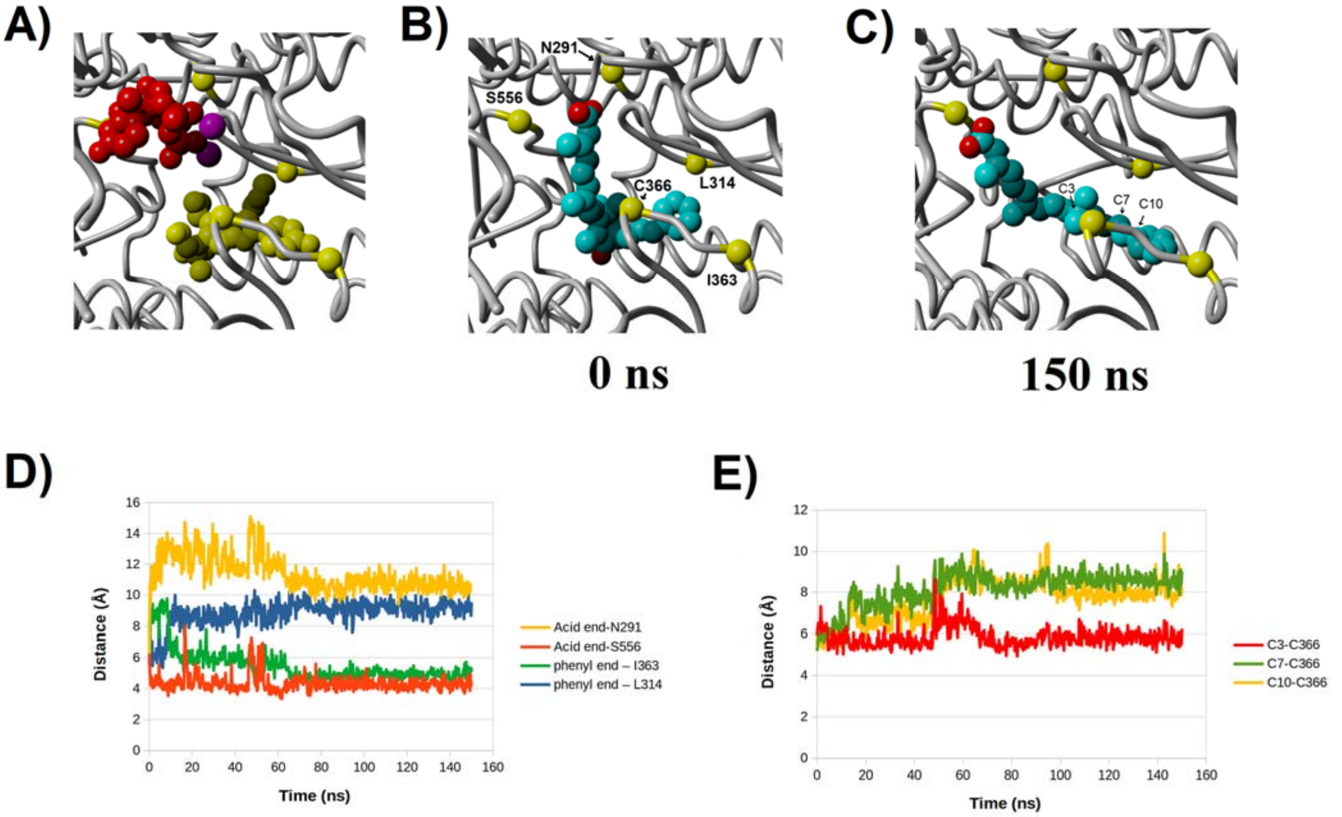

Figure S13: Evolution of the interaction of 3-cinnamyl-4-oxoretinoic acid with NS5B polymerase. For ease of comparison, the structures shown in panels A, B, and C are depicted after superposing them on each other, so that they have precisely the same orientation. A) polymerase bound to sofosbuvir (red) from PDB:4WTF. The inhibitor HCN-796 (from PDB:3FQK) is shown in yellow; B) Initial docking pose of 3-cinnamyl-4-oxoretinoic acid (ball-and-sticks model). Several Cα atoms, whose distances to the ligand are shown in panel B, are depicted as yellow balls; C) Final snapshot of the simulation of the ligand-protein complex.; D,E) Evolution of key ligand-protein distances along the simulation. Legend refers to the atoms labeled in panels B and C.

Gniditrin

Gniditrin proved to be the weakest binder, as its position relative to the active site was the least stable of all (Figure S10). It initially barely overlaps the position where the product chain will eventually grow, and as the simulation progresses even this limited overlap is lost and the molecule seemed to be on the verge of separating when the simulation ended (at 150 ns). Although this result is consistent with its poor VINA docking score, such a poor binding was not expected due to its favorable PLP.ChemScore (Table 2)

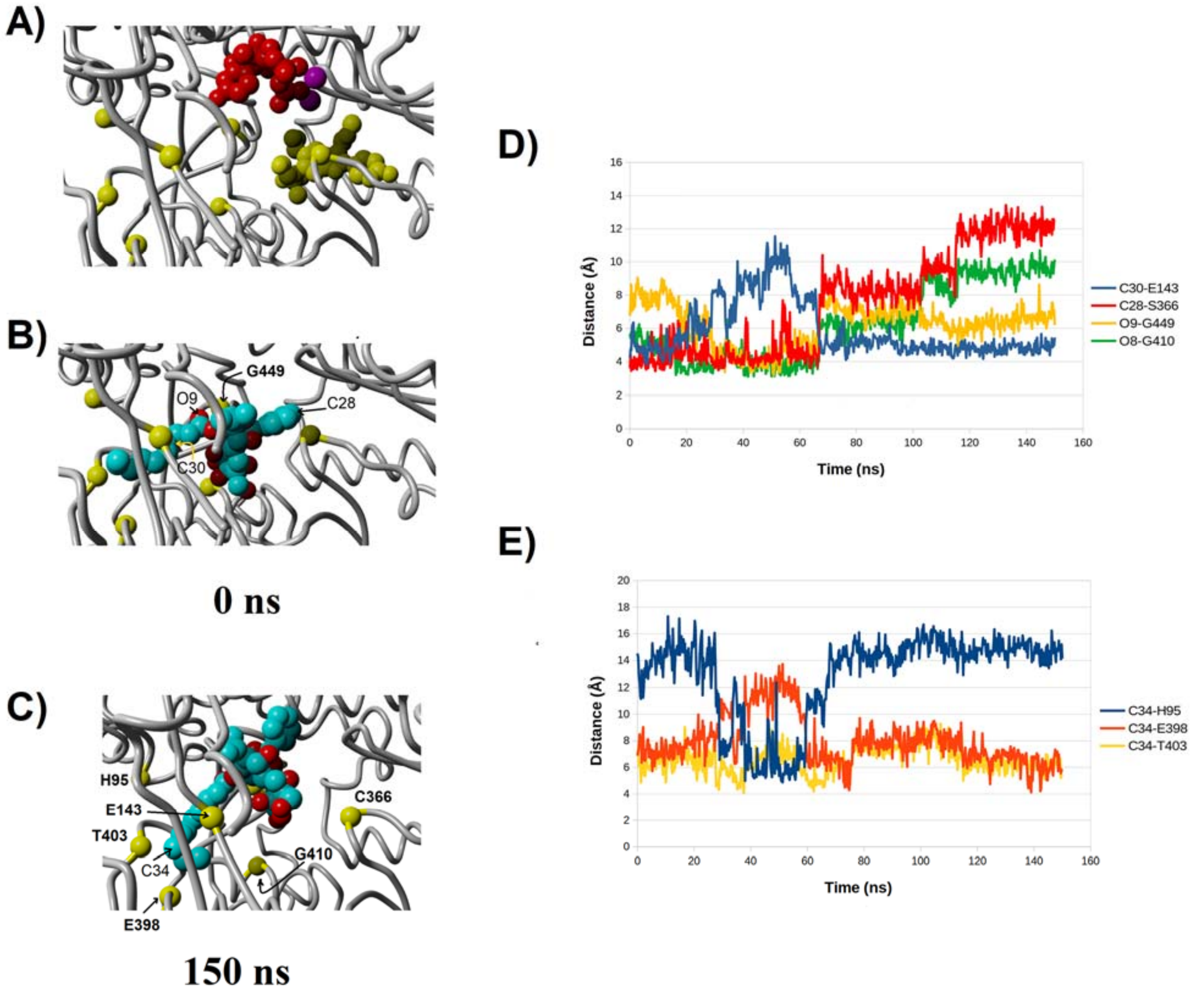

Figure S14: Evolution of the interaction of gniditrin with NS5B polymerase. For ease of comparison, the structures shown in panels A, B, and C are depicted after superposing them on each other, so that they have precisely the same orientation. A) polymerase bound to sofosbuvir (red) from PDB:4WTF. The inhibitor HCN-796 (from PDB:3FQK) is shown in yellow; B) Initial docking pose of gniditrin (ball-and-sticks model). Several Cα atoms, whose distances to the ligand are shown in panel B, are depicted as yellow balls; C) Final snapshot of the simulation of the ligand-protein complex.; D,E) Evolution of key ligand-protein distances along the simulation. Legend refers to the atoms labeled in panels B and C.
